# A Genome-Wide Copy Number Variant Study of Suicidal Behavior

**DOI:** 10.1371/journal.pone.0128369

**Published:** 2015-05-26

**Authors:** Jeffrey A. Gross, Alexandre Bureau, Jordie Croteau, Hanga Galfalvy, Maria A. Oquendo, Fatemeh Haghighi, Chantal Mérette, Ina Giegling, Colin Hodgkinson, David Goldman, Dan Rujescu, J. John Mann, Gustavo Turecki

**Affiliations:** 1 McGill Group for Suicide Studies, Douglas Mental Health University Institute, Montreal, Quebec, Canada; 2 Centre de recherche de l’Institut Universitaire en Santé Mentale de Québec, Université Laval, Quebec City, Quebec, Canada; 3 Division of Biostatistics, Department of Psychiatry, Columbia University, New York, New York, United States of America; 4 Molecular Imaging and Neuropathology Division, Department of Psychiatry, Columbia University, New York, New York, United States of America; 5 Laboratory of Neurogenetics, National Institute on Alcohol Abuse and Alcoholism, National Institutes of Health, Rockville, Maryland, United States of America; 6 Psychiatric Clinic, Martin-Luther-Universität, Halle, Saxony-Anhalt, Germany; Technische Universität Dresden, Medical Faculty, GERMANY

## Abstract

Suicide and suicide attempts are complex behaviors that result from the interaction of different factors, including genetic variants that increase the predisposition to suicidal behaviors. Copy number variations (CNVs) are deletions or duplications of a segment of DNA usually larger than one kilobase. These structural genetic changes, although quite rare, have been associated with genetic liability to mental disorders, such as autism, schizophrenia, and bipolar disorder. No genome-wide level studies have been published investigating the potential role of CNVs in suicidal behaviors. Based on single-nucleotide polymorphism array data, we followed the Penn-CNV standards to detect CNVs in 1,608 subjects, comprising 475 suicide and suicide attempt cases and 1,133 controls. Although the initial algorithms determined the presence of CNVs on chromosomes 6 and 12 in seven and eight cases, respectively, compared with none of the controls, visual inspection of the raw data did not support this finding. Furthermore we were unable to validate these findings by CNV-specific real-time polymerase chain reaction. Additionally, rare CNV burden analysis did not find an association between the frequency or length of rare CNVs and suicidal behavior in our sample population. Although our findings suggest CNVs do not play an important role in the etiology of suicidal behaviors, they are not inconsistent with the strong evidence from the literature suggesting that other genetic variants account for a portion of the total phenotypic variability in suicidal behavior.

## Introduction

Suicide and related behaviors are major public health problems that account for about one million deaths worldwide each year and impose a heavy burden on health services [1]. They are complex conditions, believed to result from the interaction of predisposing or distal factors, and more immediate factors or stressors [2–4]. Genes play an important role as predisposing factors, based on results from family, twin, and adoption studies [5]. More specifically, suicidal behavior in relatives of suicides is more common than in relatives of healthy controls, and this familial aggregation is only partially explained by liability to mental disorders, including major depressive disorder (MDD) [6, 7]. Twin and adoption studies suggest that familial aggregation of suicidal behavior is, in part, explained by genetic factors that contribute to distal predisposing or protective factors that comprise the diathesis for suicidal behavior. For instance, there is greater concordance of suicidal behavior in full siblings than in half-siblings of suicide attempters [8], and aggressive traits and mood disorders are transmitted in linkage with suicidal behavior [6]. Consistent with adoption data, monozygotic twins have higher concordance rates for suicidal behavior than dizygotic twins [9], and epidemiologically representative studies suggest that genes account for about 55% of the phenotypic variance in serious suicide attempts [10]. While these studies have consistently suggested that genetic factors contribute to the predisposition to suicidal behaviors, molecular studies have not yet identified specific genes. Indeed, many candidate gene studies and several genome-wide linkage and association studies have been conducted to date, some of which controlled for psychiatric phenotypes, and have produced largely unreplicated results.

Copy number variations (CNVs) are deletions or duplications of a segment of DNA, usually larger than 1 kilobase (kb). They constitute a form of genetic variation similar to other genetic variants, such as sequence repeats and insertion/deletions. CNVs, although quite rare, have been associated with many illnesses, including psychiatric disorders. In particular, CNVs have been observed in *de novo* cases of schizophrenia [11, 12], autism spectrum disorder [13], mental retardation [14], bipolar disorder [15, 16], MDD [17, 18], and other neurodevelopmental and neuropsychiatric disorders. With respect to suicidal behaviors, only a secondary analysis of suicide attempters was performed in a sample collected to investigate antidepressant response in MDD [19]. Here, we report findings from a study investigating the association of CNVs with suicidal behavior, including suicide and nonfatal suicide attempts, in a sample of 1,608 subjects, comprising 475 cases and 1,133 controls. Our study does not suggest that CNVs associate with suicidal behavior.

## Methods and Materials

### Subjects

Subjects included in this study were 1,608 individuals (475 cases and 1,133 controls) selected from a total sample of 2,382 unrelated individuals of Caucasian descent based on quality control procedures as described below. Subjects were recruited from three sites (New York, USA; Montreal, Canada; Munich, Germany) between 1991 and 2011.

Cases consisted of those subjects who either died by suicide or attempted suicide, where a suicide attempt was defined as a self-injurious act during which the individual had, at least, partial intent to end his/her life. The number, method, and medical damage of past suicide attempts for live subjects were recorded on the Columbia Suicide History Form. Suicidal ideation for the USA and Canadian subjects was measured using the Scale for Suicidal Ideation [20]. Diagnosis of major psychiatric disorders in suicides was determined using the Structured Clinical Interview for DSM (SCID) I by means of a validated psychological autopsy method, as previously described [21].

Controls from the Munich site were randomly selected from the general population of Munich, Germany, and were contacted by mail. Controls from the New York and Montreal sites were solicited through advertising. The Montreal sample was composed of French Canadians, whereas the New York sample was composed of Europeans of any origin. Controls were assessed by psychiatrists or clinical psychologists and evaluated using the SCID NP version and the SCID II. In this study, we included depressed controls and non-psychiatric controls. The latter were free of axis I diagnoses, cluster B personality disorder, substance use disorder and lifetime history of a suicide attempt. Depressed controls were individuals who did not have histories of suicide attempts, but met criteria for MDD.

The coroner or medical examiner determined cause of death for deceased subjects. Biological samples were obtained from the Medical Examiner’s Office in accordance with local regulations.

The Douglas Institute Research Ethics Board, the New York State Psychiatric Institute Institutional Review Board, and the Board of the Ludwig-Maximilians-University Munich, Germany approved this study and written informed consent was provided by all subjects or next-of-kin.

### Quality Control (QC)

Subjects and controls were genotyped using the Illumina Omni1-Quad Beadchip. Initial QC procedures on the genotyping data were performed using PLINK [22]. For those subjects who passed this initial QC, single-nucleotide polymorphisms (SNPs) were filtered out of the analysis if they had call rates below 95%, minor allele frequency < 0.01, or Hardy-Weinberg Equilibrium with p < 0.0001. Samples with ambiguous sex, genotyping call completeness <95%, and duplicated individuals were excluded from the analyses. Five Multidimensional Scaling Analysis components in PLINK and a comparison to HapMap Phase 3 populations were used to exclude individuals of non-European ancestry. Logistic regression with an additive model adjusted for the first five principal components was used to test the genotype-suicidal behavior association. The association test was repeated separating suicide attempt and completed suicide as outcomes. After initial QC and each of these filtering steps, 1,810 subjects remained out of the 2,382 total sample.

CNV detection was performed using the Penn-CNV program [23] (http://www.openbioinformatics.org/penncnv/ and http://www.openbioinformatics.org/penncnv/penncnv_annotation.html) on the 1,810 subjects remaining after filtering, as described above. Additionally, the Penn-CNV GC content correction was implemented, and subjects with greater than 50 CNVs spanning at least 30 probes were removed. After GC content correction, a total of 202 were removed, resulting in 1,608 subjects remaining for CNV analyses. Of the 1,608 subjects (838 males and 770 females), 475 were cases with suicidal behavior (199 suicide attempters and 276 suicides) and 1,133 were controls. Additional information on the 1,608 subjects can be found in Table 1.

**Table 1:** Demographic information on subjects.

	Mean Age (years)	Sex (M/F)
**Controls**	**38.6**	**555/578**
Alive	37.1	452/551
Dead	49.8	103/27
**Cases**	**44.4**	**283/192**
Suicide Attempters	42.7	79/120
Suicide Completers	45.6	204/72

Table 1 shows the mean ages and the ratio of males (M) to females (F) for all 1608 subjects used in the CNV analyses, separated by phenotype.

### Data Analysis

#### CNV detection

Only the CNVs comprising at least 30 probes from the array were retained, as reliability of CNV calls is directly related to the number of probes contained within the CNV. Additionally, Penn-CNV findings were verified by detecting CNVs using circular binary segmentation (CBS) [24] on the same set of subjects to assess our results for consistency. Two analyses were performed for all comparisons between cases and control: the first contained all controls, regardless of the presence of MDD, while the second excluded the controls with a history of MDD. As similar results were obtained in both analyses, the results presented include all control samples. Power calculations can be found in Table 2.

**Table 2:** Power calculations for global CNV detection.

Frequency	Odds Ratio	Power
0.01	2.0	0.37
0.01	2.5	0.60
0.05	1.5	0.45
0.05	2.0	0.90
0.10	1.4	0.52
0.10	1.5	0.69
0.20	1.2	0.29
0.20	1.3	0.52

Table 2 shows the combinations of frequencies and odds ratios that were selected based on the principle that highly deleterious mutations, being those with high odds ratios, are more likely to be selected out of a population. As such, their frequencies would tend to remain low. Similarly, less deleterious mutations are likely to become more frequent in a given population.

#### Burden Analysis

Rare CNV segments were defined either based on their frequency or their size. CNV segments with population frequencies of 0.1%, ≤0.5%, or ≤1.0%, or sizes of ≥100kb, ≥200kb, or ≥500kb were analyzed [16, 18, 25–27]. To avoid single outlier individuals driving group effects, we considered a maximum of 20 rare CNVs per individual, as this represented the number where the frequency was close to zero. A logistic regression analysis of the presence of a CNV (deletions and duplications combined) at each segment of the genome, which was defined by changes in Penn-CNV-inferred copy number in at least one subject, was performed and was adjusted for assay batch effects. Random permutations of cases and controls were performed to compute two-sided, genome-wide p-values.

#### CNV analyses

Analyses were performed using the entire population, without differentiating phenotypes of suicidal behavior, and then by stratifying the cases based on suicidal behavior, where suicides were separated from suicide attempters during analysis. Age and sex were set as covariates and did not alter the results.

### Validation

#### Subject selection

Cases containing the CNVs were all from the Montreal site. As such, validations of statistically significant CNV segments were conducted in all cases having CNVs, and in a sample of equal number of randomly selected controls from the Montreal site.

#### Probe selection

One TaqMan probe was selected based on genomic location (within the boundaries of the CNV and, when applicable, within a CBS CNV call) from the Life Technologies website for chromosomes 6 and another for chromosome 12 (Hs00558050_cn and Hs06360551_cn, respectively). RNase P (cat. #: 4403328) was used as a reference assay, as it only contains 2 copies. In addition, we used an individual with a confirmed deletion on chromosome 3 (Taqman assay Hs03487796_cn) as a positive control for the presence of a deletion (S1A and S1B Fig).

#### TaqMan Copy Number Assay

Following a standard Life Technologies protocol, quantitative real-time polymerase chain reaction (qRT-PCR) was run in quadruplicate, and target probes (FAM-MGB) were multiplexed with RNase P reference assay (VIC-TAMRA). 20ng of DNA per subject (2.0ul) per well were mixed with 5.0ul of 2x TaqMan Mastermix, 0.5ul of each 20x TaqMan assays (target and reference), and 2.0ul of deionized H_2_O for a total volume of 10.0ul per well. The plate was run on the 7900HT Real-Time PCR System using Absolute Quantification and the 9600 emulation setting. The parameters were: 1 cycle of 10 minutes at 95°C followed by 40 cycles of 95°C for 15 seconds and 60°C for 60 seconds. The plate was then analyzed using SDS2.4 with a manual CT threshold of 0.2. All data analyses were performed using the CopyCaller software version 2.0 (Life Technologies), as per manufacturer’s instructions.

## Results

**Fig 1:**
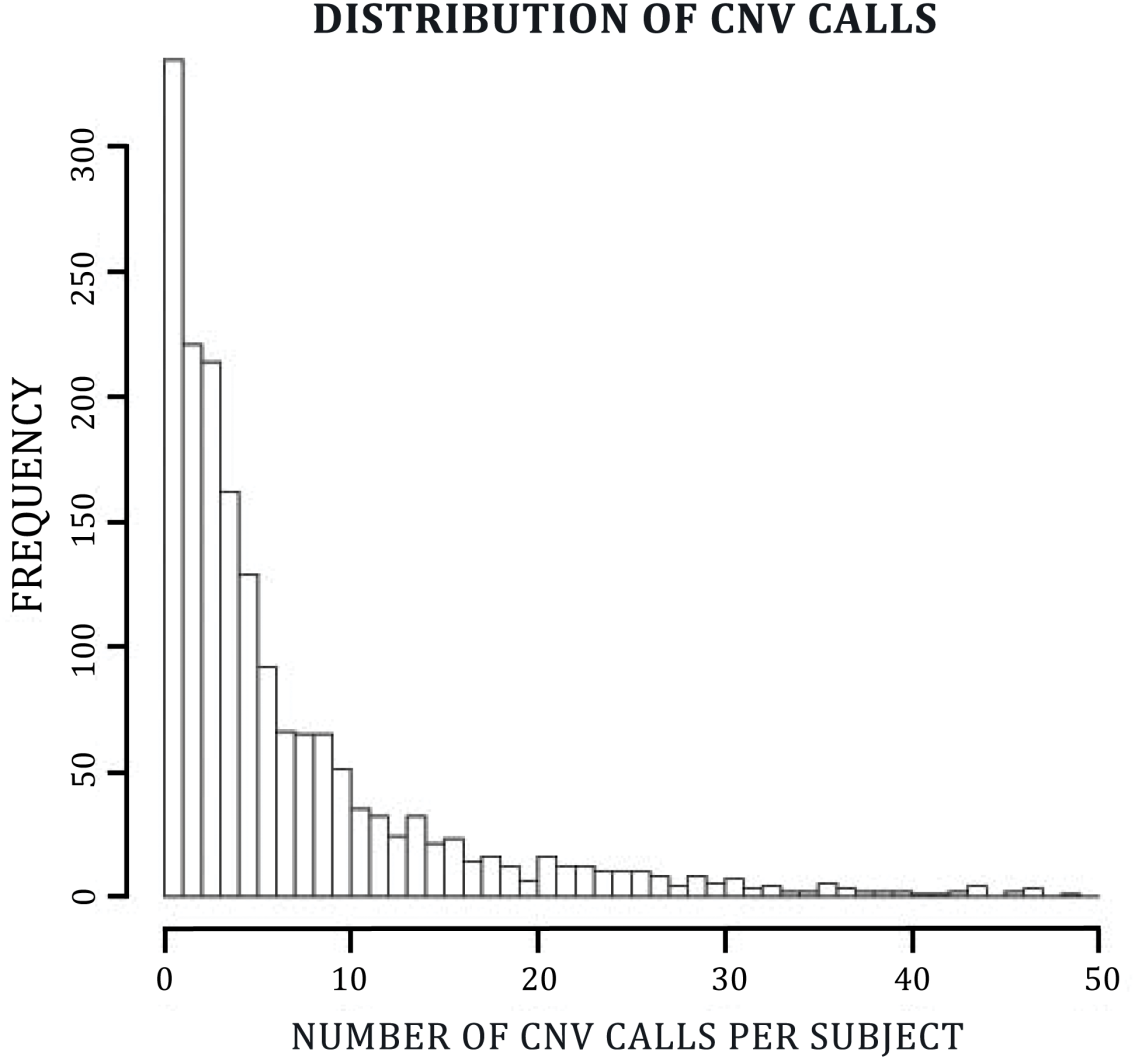
Distribution of CNV calls per subject. The figure represents a histogram with the frequency of CNV calls, with at least 30 probes, per subject.

In a population of 475 cases with suicidal behavior and 1,133 controls, we identified a total of 10,742 CNV calls that contained a minimum of 30 probes spanning the length of the CNV. Fig 1 shows the distribution of CNV calls per subject (Fig 1). Within most of the CNV segments, regardless of group, few individuals had a copy number differing from two. Specifically, of 50 CNV segments observed uniquely in cases, only 18 were present in five or more individuals. Similarly, of 272 CNV segments observed among controls, only 59 were present in five or more individuals. Of the 322 CNV segments observed exclusively in either cases or controls, no CNV was present in more than 9 subjects.

Analysis of frequency distribution of CNVs yielded no CNV segments with frequencies that were different between nonfatal suicide attempters and controls, whereas a comparison of suicides to controls identified two significantly different CNV segments (corrected p < 0.05; n = 7 for chromosome 6 and n = 8 for chromosome 12). One CNV segment comprising 166,986 bases was found on chromosome 12q12 (40,549,708–40,716,694) and contained the gene coding for leucine-rich repeat kinase 2 (LRRK2). The other CNV segment comprising 136,237 bases was found on chromosome 6p22.2 (26,150,806–26,287,043) and included a large histone H1 gene cluster. Notably, a global comparison of all cases, including attempters and suicides versus controls, yielded similar significant results for chromosomes 6 and 12. Neither the chromosome 6 nor the chromosome 12 CNV segment was present in any of the 1,133 controls analyzed in this study. Neither of the two CNV segments has been previously reported in studies investigating psychiatric phenotypes.

As literature in neuropsychiatric disorders suggests that rare CNVs may be causative for complex diseases [25–27], we performed a rare CNV burden analysis to determine if there were more rare CNV segments in individuals with suicidal behavior as compared to controls. When performing the burden analysis, we conducted logistic regressions of phenotype on the adjusted number of CNVs. We found the increase in suicide risk with each additional rare CNV was positive, but non-significant (p > 0.05). Results for analyses using varying frequencies and sizes of CNVs can be found in Table 3. When comparing the mean number of rare CNVs between cases and controls, the former had an average of 1.2 times the number of CNVs of the controls (two-sided p > 0.05).

**Table 3:** CNV burden analyses using multiple parameters.

	Total	p value^a^	OR^b^	Power
**Frequency**				
0.1%	1,363	0.18	1.07	0.53
≤ 0.5%	3,134	1.0	1.01	0.02
≤ 1.0%	3,987	1.0	1.02	0.13
**Size**				
≥ 100kb	3,289	1.0	1.03	0.10
≥ 200kb	1,251	1.0	1.09	0.19
≥ 500kb	192	0.91	0.746	0.20

Table 3 shows the effect of CNV burden based on the frequencies or sizes of the CNVs.

^a^p values are Bonferroni corrected

^b^OR: Odds Ratios for each additional CNV

**Fig 2:**
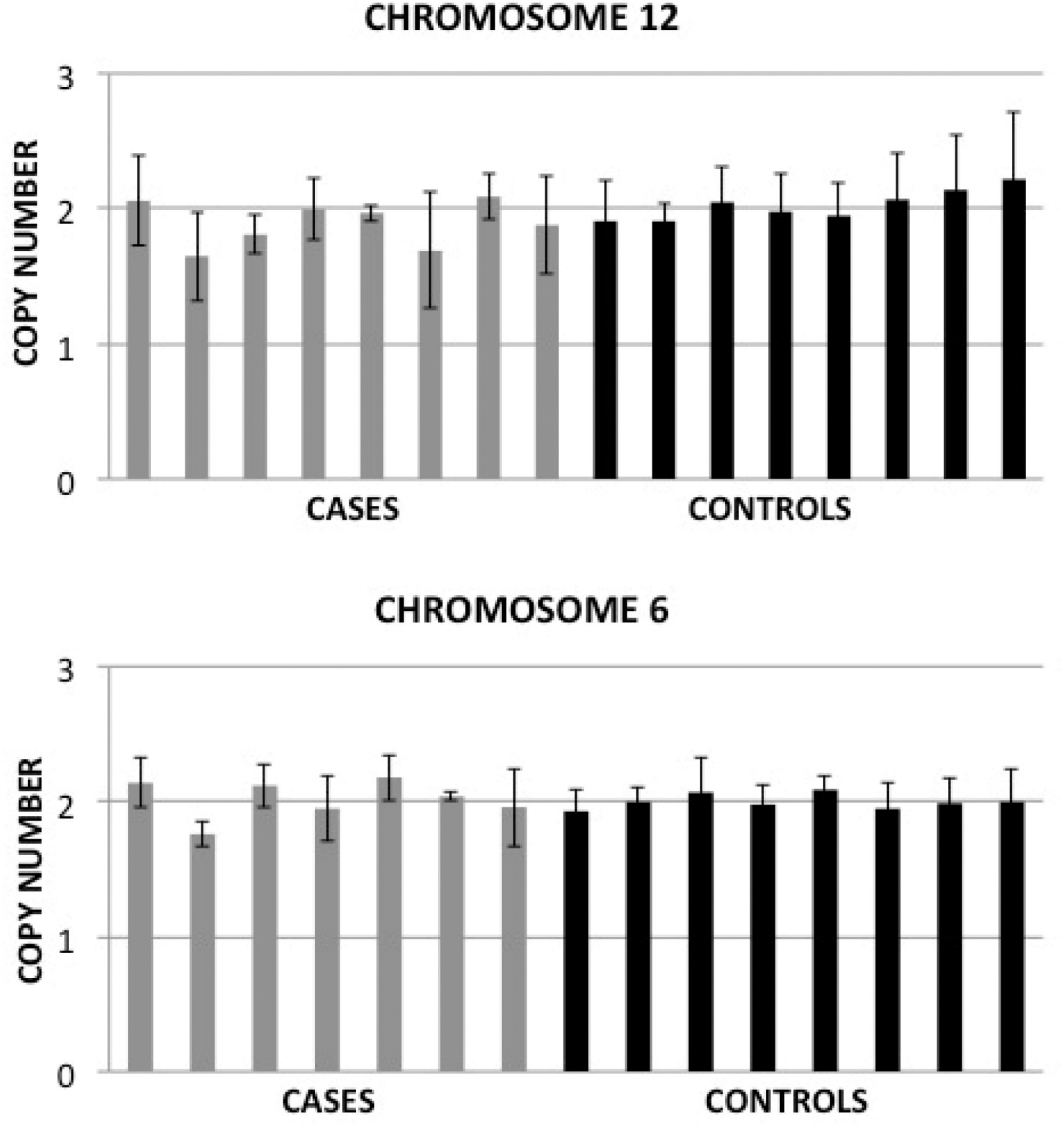
Validation of CNV call from SNP-array data does not confirm the presence of a CNV on Chromosomes 6 and 12. Seven (chromosome 6) or eight (chromosome 12) cases (grey) and eight controls (black) were probed with a TaqMan Copy Number Assay to determine the presence of a CNV. This probe was normalized to the RNase P endogenous control. Data shown represents the calculated copy number ± the copy number range, as determined by CopyCaller 2.0.

For validation, we used qRT-PCR TaqMan copy number assays, testing one genomic region that was common among all individuals carrying the 12q12 CNV segment, and, similarly, a genomic region common among all individuals carrying the 6q22.2 CNV segment. We were unable to validate the presence of a CNV on either chromosome (Fig 2). The qRT-PCR analysis determined with greater than 99% confidence that all subjects, cases and controls alike, contained two copies of the region of interest.

## Discussion

CNVs represent deletions or duplications of a segment of DNA, usually larger than 1 kb, and have previously been linked to neurodevelopmental disorders, such as autism and schizophrenia. Our study is the first genome-wide CNV analysis where suicide behavior was the primary criterion for recruitment. A previous CNV study was a secondary analysis of suicidal behavior in subjects with major depressive disorder recruited for a large trial of antidepressant treatment [19]. This study found several loci that did not reach genome-wide significance. An additional study was a case report generated from a study of bipolar patients and described a CNV in the 5-HTR1A gene in a single individual with history of suicide attempts [28]. Finally, a third study was of suicides and focused on CNVs in discrete genomic loci coding for CYP2D6 and CYP2C19 [29].

In our sample, we did not find evidence for the effects of CNVs previously reported in studies investigating patients with schizophrenia, developmental disorders, or mood disorders [30], nor for effects of CNVs reported in the studies of suicidal behavior discussed above [19, 28, 29]. Our initial genome-wide analysis identified one CNV segment on chromosome 12q12 and one CNV segment on chromosome 6p22.2, respectively in eight and seven cases, and no controls. The CNV segment on chromosome 12q12 contained the LRRK2 gene. Point mutations in LRRK2 are considered the most significant contributor to the pathogenesis of Parkinson’s disease, although the exact molecular mechanism has yet to be established [31–33]. Additionally, a specific variant of LRRK2 may have a protective role in patients with sporadic Alzheimer’s disease [34]. Regardless, LRRK2 may be of interest for suicidal behaviors as this gene is thought to increase the transcriptional activity of genes, either by inhibiting the action of Argonaute in the microRNA activity pathway [35] or by targeting ribosomal proteins [36]. Therefore, it may regulate the activity of genes involved in the neurobiology of suicidal behavior.

The CNV segment on chromosome 6p22.2 comprised a histone H1 gene cluster. Histones in the H1 family play a role in both the core histone complex, known as the nucleosome, and linker histones. Importantly, histones are susceptible to post-translational modifications, with potential effects on their ability to package the DNA. Changes in the interactions between histone complexes and DNA strands ultimately lead to changes in the expression of the genes encoded by the given segment of DNA, without altering the underlying DNA sequence. This type of epigenetic process has been a key focus of recent studies aiming to decipher the neurobiology of psychiatric disorders. Interestingly, microRNAs are also considered key to the role of epigenetics on disease pathogenesis. Given known effects of LRRK2 on Argonaute, one could hypothesize that an interaction between histone-related epigenetic alterations and LRRK2-induced microRNA dysfunctions may be related to the etiology of suicidal behavior.

Although our CNV investigation provided two interesting results, we were unable to confirm the presence of a CNV at these loci by an independent, low-throughput method (qRT-PCR). Array-based methodologies are less reliable, whereas qPCR-based assays are more sensitive and accurate [37, 38], and this may explain the lack of validation. Similarly, our inability to replicate previous findings in MDD [17], treatment-resistant MDD [18], or suicide [19, 29] is possibly a result of the large and heterogeneous sample set we investigated or of differences in the severity of the phenotypes investigated.

CNVs have been more definitively associated with neurodevelopmental conditions, particularly autism and schizophrenia, and arguably less so with mood disorders, which is the most frequent psychopathology associated with suicidal behavior. Both the previous genome-wide CNV study investigating suicidal behavior [19] and our study failed to identify significant evidence of increased burden of rare CNVs or mean number of CNVs. Thus, if CNVs are etiologically related to suicidal behavior, they are likely to explain only a small fraction of the total genetic variability.

In summary, the present study conducted a CNV analysis in a large sample of individuals with suicidal behavior and did not uncover a validated CNV associated with this phenotype. Since both this study and previous ones suggest CNVs in suicidal behavior are rare, future research should use methods that increase the likelihood of detecting these rare variants. Although detecting CNVs from next-generation sequencing data is currently challenging [39], sequencing studies are quickly becoming more feasible and affordable, and should provide the necessary sensitivity to determine the role of CNVs in suicidal behavior.

## Supporting Information

S1 Fig Positive control for CNV validation confirms validity of genome-wide assays and validation technique. A) Raw data from genome-wide CNV analysis shows a deletion on chromosome 3. B) TaqMan Copy Number Assay confirms the presence of a deletion on chromosome 3 in an individual subject compared to 8 control subjects. This probe was normalized to the RNase P endogenous control. Data shown represents the calculated copy number ± the copy number range, as determined by CopyCaller 2.0
(PDF)

## References

[1] World Health Statistics. 2012.

[2] Turecki G, Ernst C, Jollant F, Labonte B, Mechawar N. The neurodevelopmental origins of suicidal behavior. Trends Neurosci. 2012;35(1):14–23. Epub 2011/12/20. 10.1016/j.tins.2011.11.008 . 22177979

[3] van Heeringen K, Mann JJ. The Neurobiology of Suicide. The Lancet Psychiatry. 2014;1(1):63–72. 26360403 10.1016/S2215-0366(14)70220-2

[4] Oquendo MA, Perez-Rodriguez MM, Poh E, Sullivan G, Burke AK, Sublette ME, et al Life events: a complex role in the timing of suicidal behavior among depressed patients. Mol Psychiatry. 2014;19(8):902–9. 10.1038/mp.2013.128 24126928 PMC3988274

[5] Ernst C, Mechawar N, Turecki G. Suicide neurobiology. Prog Neurobiol. 2009;89(4):315–33. Epub 2009/09/22. 10.1016/j.pneurobio.2009.09.001 . 19766697

[6] McGirr A, Alda M, Seguin M, Cabot S, Lesage A, Turecki G. Familial aggregation of suicide explained by cluster B traits: a three-group family study of suicide controlling for major depressive disorder. Am J Psychiatry. 2009;166(10):1124–34. 10.1176/appi.ajp.2009.08111744 . 19755577

[7] Brent DA, Bridge J, Johnson BA, Connolly J. Suicidal behavior runs in families. A controlled family study of adolescent suicide victims. Arch Gen Psychiatry. 1996;53(12):1145–52. . 8956681 10.1001/archpsyc.1996.01830120085015

[8] Tidemalm D, Runeson B, Waern M, Frisell T, Carlstrom E, Lichtenstein P, et al Familial clustering of suicide risk: a total population study of 11.4 million individuals. Psychological medicine. 2011;41(12):2527–34. 10.1017/S0033291711000833 21733212 PMC3207221

[9] Turecki G. Suicidal behavior: is there a genetic predisposition? Bipolar disorders. 2001;3(6):335–49. . 11843783 10.1034/j.1399-5618.2001.30608.x

[10] Statham DJ, Heath AC, Madden PA, Bucholz KK, Bierut L, Dinwiddie SH, et al Suicidal behaviour: an epidemiological and genetic study. Psychological medicine. 1998;28(4):839–55. . 9723140 10.1017/s0033291798006916

[11] Ingason A, Rujescu D, Cichon S, Sigurdsson E, Sigmundsson T, Pietilainen OP, et al Copy number variations of chromosome 16p13.1 region associated with schizophrenia. Mol Psychiatry. 2011;16(1):17–25. Epub 2009/09/30. 10.1038/mp.2009.101 19786961 PMC3330746

[12] International Schizophrenia C. Rare chromosomal deletions and duplications increase risk of schizophrenia. Nature. 2008;455(7210):237–41. Epub 2008/08/01. 10.1038/nature07239 . 18668038 PMC3912847

[13] Szatmari P, Paterson AD, Zwaigenbaum L, Roberts W, Brian J, Liu XQ, et al Mapping autism risk loci using genetic linkage and chromosomal rearrangements. Nat Genet. 2007;39(3):319–28. Epub 2007/02/27. 10.1038/ng1985 . 17322880 PMC4867008

[14] Berkel S, Marshall CR, Weiss B, Howe J, Roeth R, Moog U, et al Mutations in the SHANK2 synaptic scaffolding gene in autism spectrum disorder and mental retardation. Nat Genet. 2010;42(6):489–91. Epub 2010/05/18. 10.1038/ng.589 . 20473310

[15] Malhotra D, McCarthy S, Michaelson JJ, Vacic V, Burdick KE, Yoon S, et al High frequencies of de novo CNVs in bipolar disorder and schizophrenia. Neuron. 2011;72(6):951–63. Epub 2011/12/27. 10.1016/j.neuron.2011.11.007 . 22196331 PMC3921625

[16] McQuillin A, Bass N, Anjorin A, Lawrence J, Kandaswamy R, Lydall G, et al Analysis of genetic deletions and duplications in the University College London bipolar disorder case control sample. European journal of human genetics: EJHG. 2011;19(5):588–92. Epub 2011/01/06. 10.1038/ejhg.2010.221 21206513 PMC3083610

[17] Glessner JT, Wang K, Sleiman PM, Zhang H, Kim CE, Flory JH, et al Duplication of the SLIT3 locus on 5q35.1 predisposes to major depressive disorder. PLoS One. 2010;5(12):e15463 10.1371/journal.pone.0015463 21152026 PMC2995745

[18] O'Dushlaine C, Ripke S, Ruderfer DM, Hamilton SP, Fava M, Iosifescu DV, et al Rare Copy Number Variation in Treatment-Resistant Major Depressive Disorder. Biol Psychiatry. 2014 10.1016/j.biopsych.2013.10.028 . 24529801 PMC4104153

[19] Perlis RH, Ruderfer D, Hamilton SP, Ernst C. Copy number variation in subjects with major depressive disorder who attempted suicide. PLoS One. 2012;7(9):e46315 10.1371/journal.pone.0046315 23029476 PMC3459919

[20] Beck AT, Kovacs M, Weissman A. Assessment of suicidal intention: the Scale for Suicide Ideation. Journal of consulting and clinical psychology. 1979;47(2):343–52. . 469082 10.1037//0022-006x.47.2.343

[21] Kelly TM, Mann JJ. Validity of DSM-III-R diagnosis by psychological autopsy: a comparison with clinician ante-mortem diagnosis. Acta Psychiatr Scand. 1996;94(5):337–43. Epub 1996/11/01. . 9124080 10.1111/j.1600-0447.1996.tb09869.x

[22] Purcell S, Neale B, Todd-Brown K, Thomas L, Ferreira MA, Bender D, et al PLINK: a tool set for whole-genome association and population-based linkage analyses. American journal of human genetics. 2007;81(3):559–75. . 17701901 10.1086/519795 PMC1950838

[23] Wang K, Li M, Hadley D, Liu R, Glessner J, Grant SF, et al PennCNV: an integrated hidden Markov model designed for high-resolution copy number variation detection in whole-genome SNP genotyping data. Genome Res. 2007;17(11):1665–74. 10.1101/gr.6861907 17921354 PMC2045149

[24] Olshen AB, Venkatraman ES, Lucito R, Wigler M. Circular binary segmentation for the analysis of array-based DNA copy number data. Biostatistics. 2004;5(4):557–72. 10.1093/biostatistics/kxh008 . 15475419

[25] Cooper GM, Coe BP, Girirajan S, Rosenfeld JA, Vu TH, Baker C, et al A copy number variation morbidity map of developmental delay. Nat Genet. 2011;43(9):838–46. 10.1038/ng.909 21841781 PMC3171215

[26] International Schizophrenia C. Rare chromosomal deletions and duplications increase risk of schizophrenia. Nature. 2008;455(7210):237–41. 10.1038/nature07239 18668038 PMC3912847

[27] Pinto D, Pagnamenta AT, Klei L, Anney R, Merico D, Regan R, et al Functional impact of global rare copy number variation in autism spectrum disorders. Nature. 2010;466(7304):368–72. 10.1038/nature09146 20531469 PMC3021798

[28] Perlis RH, Ruderfer D, Maussion G, Chambert K, Gallagher P, Turecki G, et al Bipolar disorder and a history of suicide attempts with a duplication in 5HTR1A. Am J Psychiatry. 2012;169(11):1213–4. Epub 2012/11/07. 10.1176/appi.ajp.2012.12050592 . 23128927

[29] Zackrisson AL, Lindblom B, Ahlner J. High frequency of occurrence of CYP2D6 gene duplication/multiduplication indicating ultrarapid metabolism among suicide cases. Clinical pharmacology and therapeutics. 2010;88(3):354–9. Epub 2009/11/13. 10.1038/clpt.2009.216 . 19907421

[30] Malhotra D, Sebat J. CNVs: harbingers of a rare variant revolution in psychiatric genetics. Cell. 2012;148(6):1223–41. 10.1016/j.cell.2012.02.039 22424231 PMC3351385

[31] Ling H, Kara E, Bandopadhyay R, Hardy J, Holton J, Xiromerisiou G, et al TDP-43 pathology in a patient carrying G2019S LRRK2 mutation and a novel p.Q124E MAPT. Neurobiol Aging. 2013;34(12):2889 e5–9. 10.1016/j.neurobiolaging.2013.04.011 23664753 PMC3906605

[32] Manzoni C, Mamais A, Dihanich S, McGoldrick P, Devine MJ, Zerle J, et al Pathogenic Parkinson's disease mutations across the functional domains of LRRK2 alter the autophagic/lysosomal response to starvation. Biochem Biophys Res Commun. 2013;441(4):862–6. 10.1016/j.bbrc.2013.10.159 24211199 PMC3858825

[33] Muda K, Bertinetti D, Gesellchen F, Hermann JS, von Zweydorf F, Geerlof A, et al Parkinson-related LRRK2 mutation R1441C/G/H impairs PKA phosphorylation of LRRK2 and disrupts its interaction with 14-3-3. Proc Natl Acad Sci U S A. 2014;111(1):E34–43. 10.1073/pnas.1312701111 24351927 PMC3890784

[34] Li HL, Lu SJ, Sun YM, Guo QH, Sadovnick AD, Wu ZY. The LRRK2 R1628P variant plays a protective role in Han Chinese population with Alzheimer's disease. CNS neuroscience & therapeutics. 2013;19(4):207–15. 10.1111/cns.12062 . 23421816 PMC6493338

[35] Gehrke S, Imai Y, Sokol N, Lu B. Pathogenic LRRK2 negatively regulates microRNA-mediated translational repression. Nature. 2010;466(7306):637–41. 10.1038/nature09191 20671708 PMC3049892

[36] Martin I, Kim JW, Lee BD, Kang HC, Xu JC, Jia H, et al Ribosomal Protein s15 Phosphorylation Mediates LRRK2 Neurodegeneration in Parkinson's Disease. Cell. 2014;157(2):472–85. 10.1016/j.cell.2014.01.064 . 24725412 PMC4040530

[37] D'Haene B, Vandesompele J, Hellemans J. Accurate and objective copy number profiling using real-time quantitative PCR. Methods. 2010;50(4):262–70. 10.1016/j.ymeth.2009.12.007 . 20060046

[38] Weaver S, Dube S, Mir A, Qin J, Sun G, Ramakrishnan R, et al Taking qPCR to a higher level: Analysis of CNV reveals the power of high throughput qPCR to enhance quantitative resolution. Methods. 2010;50(4):271–6. 10.1016/j.ymeth.2010.01.003 . 20079846

[39] Samarakoon PS, Sorte HS, Kristiansen BE, Skodje T, Sheng Y, Tjonnfjord GE, et al Identification of copy number variants from exome sequence data. BMC Genomics. 2014;15:661 10.1186/1471-2164-15-661 25102989 PMC4132917

