# Supplementary figures and images for: A Genome-Wide Copy Number Variant Study of Suicidal Behavior
Source: PLoS One. 2015 May 26;10(5):e0128369. doi: 10.1371/journal.pone.0128369 (PMC4444178; doi:10.1371/journal.pone.0128369)

A)

### Chromosome 3, Deletion, logRratio

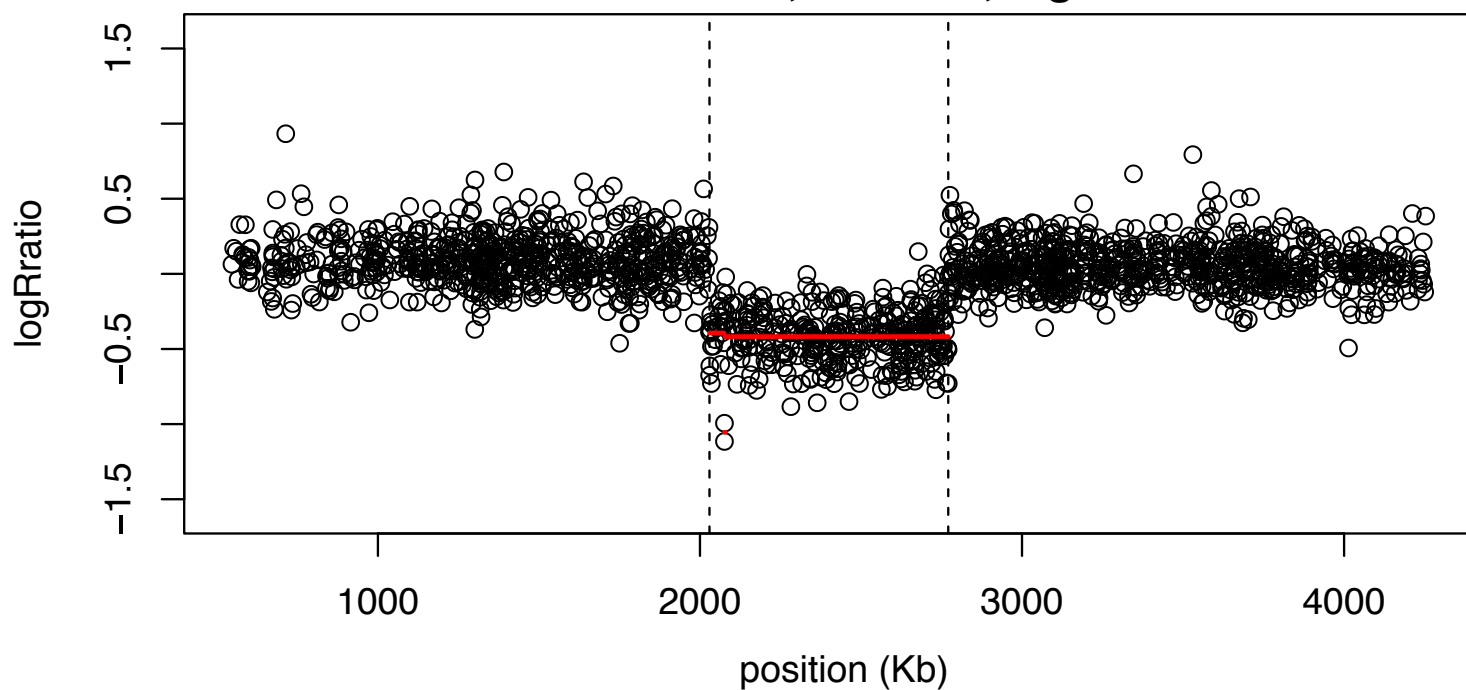

B)

### POSITIVE CONTROL FOR qPCR VALIDATION

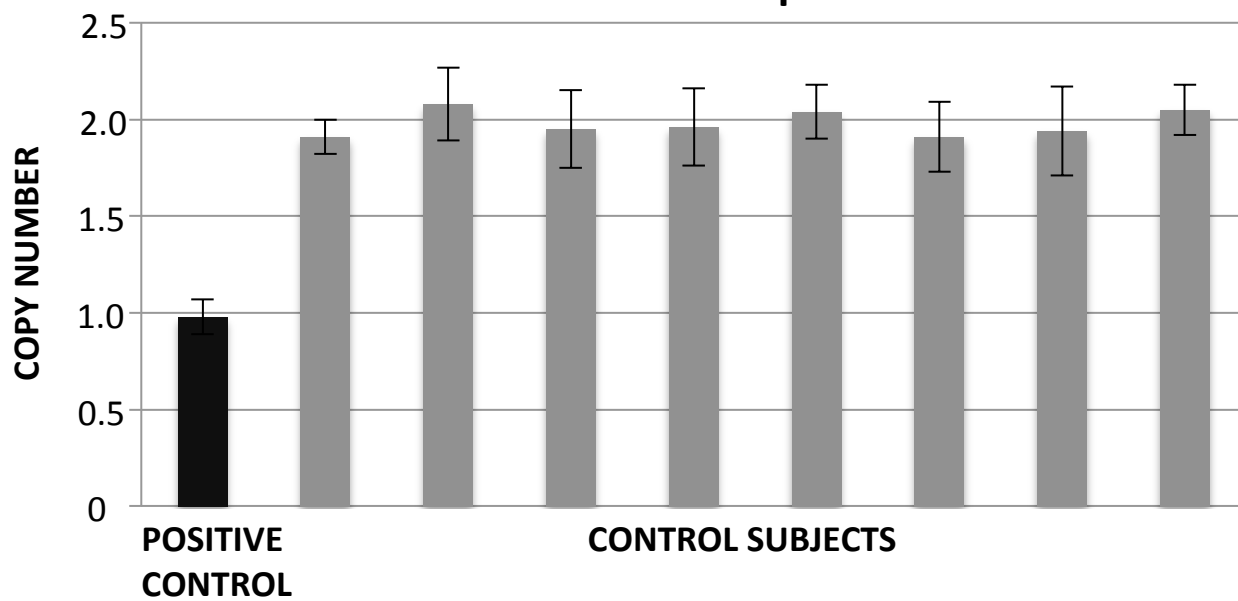

Supplement: S1 Fig — A) Raw data from genome-wide CNV analysis shows a deletion on chromosome 3. B) TaqMan Copy Number Assay confirms the presence of a deletion on chromosome 3 in an individual subject compared to 8 control subjects. This probe was normalized to the RNase P endogenous control. Data shown represents the calculated copy number ± the copy number range, as determined by CopyCaller 2.0 (PDF) [file pone.0128369.s001.pdf]
